# Multiple neurological manifestations in a patient with systemic lupus erythematosus and anti-NXP2-positive myositis: A case report

**DOI:** 10.1097/MD.0000000000025063

**Published:** 2021-03-12

**Authors:** Xiaojie Cao, Mingjie Zhang, Zhijie Lu, Congyang Li, Yanping Zeng, Jin Fan, Ke Yu

**Affiliations:** Department of Neurology, The General Hospital of Western Theater Command, Jinniu District, Chengdu, Sichuan Province, People's Republic of China.

**Keywords:** anti-NXP2 antibody, case report, myositis, neurological manifestations, systemic lupus erythematosus

## Abstract

**Rationale:**

Systemic lupus erythematosus (SLE) is a complex autoimmune inflammatory disease that frequently affects various organs. Neuropsychiatric manifestations in SLE patients, known as neuropsychiatric SLE, are clinically common. However, the principal manifestation of cranial neuropathy in patients with SLE and comorbidities is relatively rare.

**Patient concerns:**

In this report, we describe a 51-year-old Chinese woman who was admitted with a chief complaint of chronic-onset facial paresthesia, dysphagia, and choking cough when drinking water, accompanied by slurred speech, salivation, and limb weakness. The blood autoantibody test results showed that many SLE-associated antibodies were positive. Meanwhile, anti-nuclear matrix protein 2 (NXP2) antibody was strongly positive in the idiopathic inflammatory myopathy (IIM) spectrum test from the serum. Muscle biopsy indicated inflammatory infiltration of the muscle fiber stroma.

**Diagnoses:**

Taking into account the clinical manifestations and laboratory tests of the present case, the diagnosis of SLE and probable IIM was established.

**Interventions:**

Corticosteroids and additional gamma globulin were administered and the clinical symptoms were relieved during the treatment process.

**Outcomes:**

Unfortunately, the patient experienced sudden cardiac and respiratory arrest. Multiple system dysfunctions exacerbated disease progression, but in the present case, we speculated that myocardial damage resulting from SLE could explain why she suddenly died.

**Lessons:**

To our knowledge, multiple neurological manifestations in patients with SLE and anti-NXP2-positive myositis are rare. Note that SLE is still a life-threatening disease that causes multiple system dysfunctions, which requires increasing attention.

## 1 Introduction

Systemic lupus erythematosus (SLE) is a complex autoimmune inflammatory disease characterized by multiple systems and various clinical features. Neuropsychiatric manifestations in patients with SLE, known as neuropsychiatric SLE (NPSLE), are not uncommon clinically.^[1]^ However, the main manifestation of the peripheral nervous system (PNS) in patients with SLE and its comorbidities, such as rare myositis, is relatively infrequent.^[2]^ In this report, we describe multiple neurological manifestations, particularly involving the cranial nerves, in a female patient with SLE and anti*-*nuclear matrix protein 2 (NXP2)-positive myositis. We present the following case in accordance with the CARE reporting checklist.

## 2 Case presentation

A 51-year-old Chinese woman was admitted to our hospital on March 2, 2020, with a chief complaint of chronic-onset facial paresthesia, dysphagia, and choking cough when drinking water. The patient complained of blindness in her right eye since childhood. She had hyperthyroidism and received iodine-131 (I-131) therapy 10 years ago. She had hypertension for 7 years and was regularly taking antihypertensive drugs. Over the preceding 7 months, edema of her face gradually appeared. Three months before admission, her facial paresthesia, especially numbness, tingling, and burning on the forehead, cheeks and lips, started to appear. Unfortunately, she was admitted to many other hospitals with no definite diagnosis and symptom relief. These symptoms worsened. Moreover, she began showing symptoms of dysphagia and choking cough when drinking water, accompanied by slurred speech, salivation, and limb weakness. She had no obvious family, psychosocial or genetic history.

On the day of admission, physical examination revealed facial edema, bulging eyes, and thyroid enlargement. Her neurological examination revealed absence of bilateral corneal reflex, bilateral peripheral facial paralysis, weakness of the bilateral pharyngeal reflex, dysphagia, choking cough when drinking water and dysarthria. The muscle strength of the proximal limbs was level 4 (0–5), and the distal limbs was level 5. Superficial facial sensation decreased. The bilateral Babinski's signs were positive. There were no sensory deficits in the 4 limbs, and the tendon reflexes were normal.

Laboratory data showed increased levels of glutamic-pyruvic transaminase (176.8 IU/L), glutamic-oxalacetic transaminase (499.1 IU/L), thyroid-stimulating hormone (14.777 mIU/L), anti-thyroid peroxidase antibody (>1300.00 U/mL), anti-thyroglobulin antibody (178.80 U/mL), creatine kinase (CK, 18975.2 IU/L), and lactic dehydrogenase (1006.5 IU/L), and decreased counts of platelets (78, 000 cells/μL) and lymphocytes (770 cells/μL). White blood cell count, erythrocyte sedimentation rate, and C-reactive protein levels were normal. Renal function test showed increased levels of urinary microalbumin (135.70 mg/L), urinary microalbumin/urine creatinine I (42.63 mg/mmoL), urinary microalbumin/urine creatinine II (296.06 mg/g), and 24-hour urinary protein (2.16 g/24 h), and decreased level of complement C3 (0.42 g/L). In addition, autoantibody test results showed anti-nuclear antibody (>1:3200), anti-U1 ribonucleoprotein (RNP) antibody (+++), anti-Sm antibody (+++), anti-SP100 antibody (+++), and anti-mitochondrial antibody (M2, +++). The Coombs test result was positive. Antiphospholipid antibodies tested negative. Anti-NXP2 antibody IgG was strongly positive (+++) in the idiopathic inflammatory myopathy (IIM) spectrum test from the serum. However, the paraneoplastic antibodies were negative.

Electrophysiological tests showed that the amplitudes of the bilateral median nerve compound muscle action potential decreased, and the rest of the motor and sensory nerves of the limbs were normal. On needle electromyography (EMG), the spontaneous potentials of the left thenar muscle and right paravertebral muscle were observed in the resting state. During light contraction, the mean amplitudes of the right thenar muscle, right biceps brachii, and both sides of the sternocleidomastoid muscle increased. The blink reflex of this patient was the absence of R1 combined with delayed R2 and R2’ responses. Color Doppler ultrasound of the thyroid revealed diffuse lesions. Muscle biopsy indicated inflammatory infiltration of the muscle fiber stroma. Cranial magnetic resonance imaging (MRI) revealed some sporadic lacunar infarctions. Digital x-ray images of the upper limbs and chest did not show calcinosis. There were no findings suggesting malignant tumors and special infections, such as HIV and syphilis, in the systemic screening tests. Lumbar puncture and skin biopsy were not performed because the patient refused to undergo these procedures.

The patient was initially diagnosed with SLE based on 4 clinical findings (renal, neurologic, lymphopenia, and thrombocytopenia) and 4 immunological items (anti-nuclear antibody, anti-Sm antibody, low complement, and direct Coombs test) in the Systemic Lupus International Collaborating Clinics (SLICC) classification criteria for SLE.^[3]^ Meanwhile, accumulating 17 points in the updated classification criteria for SLE from the European League Against Rheumatism/American College of Rheumatology (EULAR/ACR) also confirmed the diagnosis (Table 1).^[1]^ The SLE disease activity index 2000 (SLEDAI-2K) score was 19 points, indicating severe activity.^[4]^ The presense of multiple neurological manifestations supported the classification of NPSLE.^[5]^ Furthermore, in light of clinical manifestations and muscle biopsy, myopathy needs to be considered. According to IIM classification criteria from EULAR/ACR, the total aggregated score was 8.3 points, including muscle biopsy, corresponding to probable IIM (Table 2).^[6]^ Without skin manifestations, the present case was classified as polymyositis (PM). Interestingly, strongly positive anti-NXP2 antibodies, common in dermatomyositis (DM), were detected in the patient's serum.

**Table 1:** SLE classification criteria from EULAR/ACR 2019.

Entry criterion
Antinuclear antibodies at a titer of ≥1:80 on HEp-2 cells or an equivalent positive test (ever)

Additive criteria^∗^			
Clinical domains and criteria	Weight	Immunology domains and criteria	Weight
*Constitutional*		*Antiphospholipid antibodies*	
Fever	2	Anti-cardiolipin antibodies OR	2
*Hematologic*		Anti-β2GP1 antibodies OR	
Leukopenia	3	Lupus anticoagulant	
Thrombocytopenia	4	*Complement proteins*	
Autoimmune hemolysis	4	Low C3 OR low C4	3
*Neuropsychiatric*		Low C3 AND low C4	4
Delirium	2	*SLE-specific antibodies*	
Psychosis	3	Anti-dSDNA antibody OR	6
Seizure	5	Anti-Smith antibody	
*Mucocutaneous*			
Non-scarring alopecia	2		
Oral ulcer	2		
Subacute cutaneous OR discoid lupus	4		
Acute cutaneous lupus	6		
*Serosal*			
Pleural or pericardial effusion	5		
Acute pericarditis	6		
*Musculoskeletal*			
Joint involvement	6		
*Renal*			
Proteinuria >0.5 g/24 h	4		
Renal biopsy Class II or V lupus nephritis	8		
Renal biopsy Class III or IV lupus nephritis	10		

EULAR/ACR = European League Against Rheumatism/American College of Rheumatology, SLE = systemic lupus erythematosus.

∗ SLE classification requires at least one clinical criterion and ≥10 points.

**Table 2:** IIM classification criteria from EULAR/ACR 2017.

Variable	Score points^∗^ with muscle biopsy
Age of onset	
Age of onset of first symptom assumed to be related to the disease ≥18 y and < 40 y	1.5
Age of onset of first symptom assumed to be related to the disease ≥40 y	2.2
Muscle weakness	
Objective symmetric weakness, usually progressive, of the proximal upper extremities	0.7
Objective symmetric weakness, usually progressive, of the proximal lower extremities	0.5
Neck flexors are relatively weaker than neck extensors	1.6
In the legs, proximal muscles are relatively weaker than distal muscles	1.2
Skin manifestations	
Heliotrope rash	3.2
Gottron's papules	2.7
Gottron's sign	3.7
Other clinical manifestations	
Dysphagia or oesophageal dysmotility	0.6
Laboratory measurements	
Anti-Jo-1 (anti-histidyl-tRNA synthetase) autoantibody present	3.8
^†^Elevated serum levels of creatine kinase or lactate dehydrogenase or aspartate aminotransferase (ASAT/AST/SGOT) or alanine aminotransferase (ALAT/ALT/SGPT)	1.4
Muscle biopsy features—presence of:	
Endomysial infiltration of mononuclear cells surrounding, but not invading, myofibres	1.7
Perimysial and/or perivascular infiltration of mononuclear cells	1.2
Perifascicular atrophy	1.9
Rimmed vacuoles	3.1

EULAR/ACR = European League Against Rheumatism/American College of Rheumatology, IIM = idiopathic inflammatory myopathy.

∗ A score of ≥6.7 and ≥8.7 with muscle biopsy corresponds to probable IIM and definite IIM, respectively.

† Serum levels above the upper limit of normal.

To determine the treatment plan, we consulted relevant departments especially the Department of Rheumatology and Immunology. On the 8th day of hospitalization, methylprednisolone pulse therapy (500 mg/day for 3 days, followed by 240 mg/day for 3 days) and gamma globulin (25 g/day for 5 days) were infused intravenously to treat severe SLE and anti-NXP2-positive myositis. Orally administered lower doses of prednisolone (60 mg/day) were administered following methylprednisolone pulse therapy.

During the treatment process, the facial swelling and paresthesia subsided gradually. Clinical symptoms, such as dysphagia, choking cough when drinking water, slurred speech, salivation, and limb weakness, were relieved. On the 13th day of hospitalization, several laboratory test results including glutamic-oxalacetic transaminase (297.4 IU/L), CK (3887.2 IU/L), and lactic dehydrogenase (927.4 IU/L) decreased in comparison with the pretreatment data.

Unfortunately, the patient experienced sudden cardiac and respiratory arrest early in the morning on the 16th day of hospitalization. The patient eventually died despite emergency rescue efforts. The patient's family refused an autopsy. With regard to the available information, we speculated that myocardial damage resulting from SLE could explain why she died suddenly.

## 3 Discussion

SLE is a complex autoimmune inflammatory disease with multiple systems and various clinical features. Definitive diagnosis of SLE mainly depends on the clinical manifestations and specific immunological changes. The 2012 SLICC classification criteria emphasized that SLE is an autoantibody-driven clinical disease that requires at least 4 criteria, including at least 1 clinical and 1 immunologic criterion.^[3]^ In addition, biopsy-confirmed lupus nephritis in the presence of antinuclear antibodies or anti-dsDNA antibodies satisfies the classification criteria for SLE.^[3]^ The 2019 update of the EULAR/ACR classification criteria for SLE contained 1 entry criterion (positive antinuclear antibodies) and 10 additive weighted criteria.^[1]^ Patients with at least 1 clinical criterion and a score ≥10 points were classified as having SLE. Taking into account the clinical manifestations and laboratory tests of the present case, the diagnosis of SLE was established according to the classification criteria from SLICC and EULAR/ACR.

Multiple clinical manifestations in the present case indicated extensive involvement of the nervous system. In 1999, the ACR developed case definitions for 19 neuropsychiatric syndromes observed in SLE, including 12 involving the central nervous system (CNS) and 7 involving the PNS.^[5]^ However, the defining characteristics of NPSLE have remained to be questioned. In the present case, the lesions mainly involving the bilateral trigeminal (V), facial (VII), glossopharyngeal (IX), vagus nerves (X), and bilateral corticospinal tracts supported the diagnosis of NPSLE. Neuropsychiatric manifestations in SLE patients are relatively common and are mainly associated with the CNS, such as headaches, cognitive dysfunction, and psychiatric disorders.^[7]^ In contrast, the prevalence of manifestations involving PNS in patients with SLE is rare. A retrospective longitudinal study from China involving 4924 SLE patients showed that the prevalence of peripheral neuropathy (PN) was 1.5%.^[2]^ Among those patients with SLE-associated PN, subtypes including >10% were polyneuropathy, mononeuropathy, cranial neuropathy, and myasthenia gravis, respectively. Data from a newly published international cohort study indicated that the prevalence of PN in SLE was 7.6%, and PN impaired the health-related quality of life.^[8]^ These data also indicated that the most frequent cranial neuropathies involved the optic (II), auditory (VIII), and facial (VII) nerves. In contrast, cranial neuropathies showed a rapid improvement. The present case raised the possibility of damage to the other cranial nerves. The pathogenesis of SLE-PN has not yet been clarified. However, immunological reactions to nerve tissue may be an indispensable factor associated with the onset of SLE-PN.^[2]^

Considering the weakness of the proximal limbs, elevated CK and inflammatory infiltration of the muscle fiber stroma, IIM in the present case was suspected. IIMs are a group of chronic systemic autoimmune diseases characterized by symmetrical and proximal muscle weakness, elevated muscle enzymes, EMG, and histological changes. According to clinical manifestations, immunologic changes, and muscle biopsy, IIMs mainly consist of PM, DM, amyopathic dermatomyositis, immune-mediated necrotizing myopathy, and sporadic inclusion body myositis (sIBM) at present.^[6]^ In addition, adult IIM distinguishes juveniles under the age of 18 years when the first symptom onset. According to the IIM classification criteria from EULAR/ACR, the present case was only diagnosed as probable IIM and then subdivided into PM due to lack of skin manifestations.^[6]^

The titration of different myositis-related autoantibodies helps to enrich and understand the stratification and development of IIM subtypes. Based on the diagnostic value of IIMs, these myositis-related autoantibodies are classified into two categories, myositis-specific autoantibodies (MSAs), and myositis-associated autoantibodies.^[9]^ MSAs are detected exclusively in myositis and contribute to stratifying patients into specific clinical subtypes. Fortunately, in the present case, anti-NXP2 antibodies were detected in the serum and were strongly positive. Anti-NXP2 antibodies, originally termed anti-MJ, were one kind of MSAs and were first identified in childhood myositis in 1997.^[10]^ Previous data have shown that anti-NXP2 autoantibodies are present in 23% to 30.2% of juvenile DM and less than 17% of adult PM/DM.^[9,11]^ In an Argentine pediatric myositis cohort, anti-NXP2 antibodies were likely linked to muscle contractures, atrophy and significant functional compromise.^[12]^ Additionally, anti-NXP2 autoantibodies detected in DM were found to be more significantly associated with calcinosis in younger patients and cancer in older male adults.^[13,14]^ Data from a meta-analysis also showed that anti-NXP2 autoantibodies in IIM patients were associated with an increased risk of edema, muscle weakness, myalgia/myodynia, dysphagia and calcinosis.^[15]^ Except for sIBM and advanced cancer-associated myositis, patients with other types of IIMs usually have a good prognosis based on prompt diagnosis and accurate treatment. To date, there is no evidence to prove that the presence of anti-NXP2 antibody increases the risk of a poor prognosis.^[15]^

The incidence of overlap syndrome, including SLE and myositis, is low, but has receiving increasing attention. Reportedly, concurrent inflammatory myositis occurs in 6.3% of SLE patients.^[16]^ In fact, many other disorders, except SLE, can cause muscle weakness in SLE patients, making it difficult for clinicians to distinguish between them. Therefore, the diagnosis of overlap syndrome requires comprehensive analysis of multiple factors, such as clinical presentations, laboratory examinations, EMG changes, and muscle biopsy. Furthermore, anti-RNP antibodies are highly frequent in patients with myositis and SLE.^[16,17]^ Similar to the present case, SLE combined with probable IIM in the presence of additional anti-NXP2 antibodies is relatively infrequent to date. Moreover, multiple cranial neuropathies and bilateral corticospinal tract injuries are uncommon clinical manifestations. Corticosteroids and additional gamma globulin were used in the present case as first-line therapy. During the treatment process, clinical symptoms were relieved. Unfortunately, the patient experienced sudden cardiac and respiratory arrest early in the morning after treatment for 8 days. In 2014, the worldwide age-standardized mortality rate for SLE was 2.68 deaths/millions inhabitants.^[18]^ SLE can involve several organs, and in this case, the nervous system, immune system, muscle, kidney, liver, and thyroid gland have suffered damage at different levels. However, what we should not ignore is that SLE can also result in myocardial inflammation and then induce sudden cardiac death, severe arrhythmias, or end-stage heart failure.^[19]^ In addition, severe autonomic dysfunctions, such as reduction in heart rate variability and prolonged QTc interval, increase the risk for the onset of sudden cardiac death in SLE patients.^[20]^ Multiple system dysfunctions exacerbated disease progression, but in the present case, we speculated that myocardial damage resulting from SLE could explain why she died suddenly.

## 4 Conclusions

To our knowledge, multiple neurological manifestations in patients with SLE and anti-NXP2-positive myositis are relatively infrequent. Despite the more effective immunotherapy, SLE is still a life-threatening disease that causes multiple system dysfunctions. The occurrence of complications and comorbidities in SLE requires increasing attention.

## Author contributions

**Conceptualization:** Xiaojie Cao, Mingjie Zhang, Jin Fan, Ke Yu.

**Data curation:** Xiaojie Cao, Zhijie Lu, Congyang Li, Yanping Zeng.

**Formal analysis:** Mingjie Zhang, Jin Fan.

**Funding acquisition:** Xiaojie Cao, Ke Yu.

**Investigation:** Xiaojie Cao, Zhijie Lu, Congyang Li, Yanping Zeng.

**Resources:** Xiaojie Cao, Zhijie Lu, Congyang Li, Yanping Zeng.

**Supervision:** Jin Fan, Ke Yu.

**Visualization:** Xiaojie Cao, Mingjie Zhang.

**Writing – original draft:** Xiaojie Cao, Mingjie Zhang.

**Writing – review & editing:** Xiaojie Cao, Mingjie Zhang, Jin Fan, Ke Yu.
